# Effect of fluconazole on the pharmacokinetics of a single dose of fedratinib in healthy adults

**DOI:** 10.1007/s00280-022-04464-w

**Published:** 2022-08-24

**Authors:** Yizhe Chen, Ken Ogasawara, Rebecca Wood-Horrall, Mark Thomas, Michael Thomas, Bing He, Liangang Liu, Yongjun Xue, Sekhar Surapaneni, Leonidas N. Carayannopoulos, Simon Zhou, Maria Palmisano, Gopal Krishna

**Affiliations:** 1 Bristol Myers Squibb, Princeton, NJ USA; 2 PPD, Wilmington, NC USA

**Keywords:** Fedratinib, CYP3A4, CYP2C19, Drug–drug interaction, Fluconazole, Pharmacokinetics

## Abstract

**Purpose:**

Fedratinib is an orally administered Janus kinase (JAK) 2–selective inhibitor for the treatment of adult patients with intermediate-2 or high-risk primary or secondary myelofibrosis. In vitro, fedratinib is predominantly metabolized by cytochrome P450 (CYP) 3A4 and to a lesser extent by CYP2C19. Coadministration of fedratinib with CYP3A4 inhibitors is predicted to increase systemic exposure to fedratinib. This study evaluated the effect of multiple doses of the dual CYP3A4 and CYP2C19 inhibitor, fluconazole, on the pharmacokinetics of a single dose of fedratinib.

**Methods:**

In this non-randomized, fixed-sequence, open-label study, healthy adult participants first received a single oral dose of fedratinib 100 mg on day 1. Participants then received fluconazole 400 mg on day 10 and fluconazole 200 mg once daily on days 11–23, with a single oral dose of fedratinib 100 mg on day 18. Pharmacokinetic parameters were calculated for fedratinib administered with and without fluconazole.

**Results:**

A total of 16 participants completed the study and were included in the pharmacokinetic population. Coadministration of fedratinib with fluconazole increased maximum observed plasma concentration (*C*_max_) and area under the plasma concentration–time curve from time 0 to the last quantifiable concentration (AUC_0–t_) of fedratinib by 21% and 56%, respectively, compared with fedratinib alone. Single oral doses of fedratinib 100 mg administered with or without fluconazole were well tolerated.

**Conclusions:**

Systemic exposure after a single oral dose of fedratinib was increased by up to 56% when fedratinib was coadministered with fluconazole compared with fedratinib alone.

**Trial registry: Clinicaltrials.gov:**

NCT04702464.

## Introduction

Myeloproliferative neoplasms are malignancies arising from somatically mutated hematopoietic stem cells [1]. Myelofibrosis is a type of myeloproliferative neoplasm defined by megakaryocytic hyperplasia and bone marrow fibrosis and is associated with mutations in Janus kinase (JAK) 2 and dysregulation of the JAK/STAT pathway [1–3]. Patients with myelofibrosis have a poor prognosis, with median survival of between 4.4 and 5.9 years [4, 5]. In addition, treatment options for myelofibrosis are limited, with hematopoietic stem cell transplant (HSCT) being the only potentially curative treatment [6] and an option for higher-risk patients who are transplant candidates [7]. Yet HSCT is associated with morbidity and mortality, and many patients are ineligible [8].

As an alternative to HSCT, JAK inhibitors such as fedratinib and ruxolitinib have been shown to reduce symptoms and prolong survival for patients with myelofibrosis [9–12]. Fedratinib (formerly TG101348/SAR302503) is an oral kinase inhibitor with activity against wild type and mutationally activated JAK2 and FMS-like tyrosine kinase 3 (FLT3) [13]. In the double-blind Phase 3 JAKARTA study (NCT01437787), in which patients with intermediate-2 or high-risk myelofibrosis were randomly assigned to treatment with fedratinib or placebo, a greater proportion of patients treated with fedratinib reached the primary (≥ 35% reduction in spleen volume) and secondary (≥ 50% reduction in symptom score) endpoints compared with those treated with placebo [10]. Fedratinib is approved in the USA for the treatment of adult patients with intermediate-2 or high-risk myelofibrosis [13], and in the EU for the treatment of splenomegaly or symptoms of disease in adult patients with myelofibrosis who are JAK inhibitor naive or have been treated with ruxolitinib [14], both at a recommended dose of 400 mg once daily.

Use of multiple medications puts patients at increased risk for drug–drug interactions (DDIs) [15]. The majority of pharmacokinetic (PK) interactions result from inhibition of cytochrome P450 (CYP) enzymes, which are involved in the metabolism of many drugs [15, 16]. Fedratinib is metabolized in vitro by CYP3A4, and to a lesser extent by CYP2C19 and flavin-containing monooxygenases (FMOs) [13]. At 0.1 μM fedratinib, the CYP3A4 contribution to metabolism was highly variable among different hepatocyte preparations, with CYP3A4 representing 0–67% of the total CYP contribution. CYP2D6, CYP2C19, and FMOs had a low contribution to fedratinib metabolism. In healthy participants, the mean fractions of metabolism or excretion in relation to systemic clearance were estimated to be 16% for CYP2C19 (liver) and 64% for CYP3A4 (liver) following the first dose of 400 mg once-daily fedratinib, which are overall in good agreement with in vitro (hepatocytes and microsomes) data [17]. Analysis of fedratinib metabolites in the plasma of healthy adult men after an oral dose of [^14^C]-fedratinib 200 mg showed that fedratinib accounted for approximately 80% of the total circulating drug in plasma, with none of the metabolites present at > 10% of the total circulating drug levels [18]. A population PK study showed that, after oral administration, fedratinib exhibits biphasic disposition and linear, time-invariant PK in patients with myelofibrosis [19]. The terminal elimination half-life (*t*_1/2_) for fedratinib is around 114 h, and effective *t*_1/2_ is around 40 h [13, 20].

Current prescribing information recommends avoiding the use of fedratinib with dual CYP3A4 and CYP2C19 inhibitors [13, 14]. Fluconazole is an antifungal agent indicated for the treatment of candidiasis and cryptococcal meningitis [21], and is widely used for antifungal prophylaxis in adults with hematologic malignancies [22]. Fluconazole is also a potent CYP2C19 inhibitor and a moderate CYP3A4 inhibitor [21, 23, 24], and thus is an appropriate drug to assess for PK perpetrator interactions with fedratinib. Predictions from physiologically based pharmacokinetic (PBPK) simulations of coadministration of fluconazole suggest up to a fourfold increase in fedratinib exposure versus fedratinib alone [17]. The primary objective of this study was to evaluate the effect of multiple doses of fluconazole on the PK of a single dose of fedratinib in healthy adults. The secondary objective was to evaluate the safety and tolerability of a single dose of fedratinib when administered with or without fluconazole in healthy adult participants.

## Materials and methods

### Study population

This was an open-label Phase 1 study to evaluate the effect of multiple doses of fluconazole on the PK, safety, and tolerability of a single dose of fedratinib in healthy adult participants (NCT04702464). Healthy men and women aged 18–65 years with body mass index (BMI) ≥ 18 and ≤ 33 kg/m^2^ at screening were enrolled at one site in the USA. Participants were confirmed to be healthy based on medical history, physical examination, clinical laboratory test results, vital signs, and 12-lead electrocardiogram (ECG) at screening and check-in (day − 1). Aspartate aminotransferase, alanine aminotransferase, and total bilirubin must have been at or below the upper limit of the reference range on or before check-in (day − 1). Other clinical parameters and laboratory results were either within normal range or deemed not clinically significant by the investigator. Women of childbearing potential were required to have a negative pregnancy test result and to use birth control.

Exclusion criteria included a history of or screening observations consistent with clinically relevant disease, drug or alcohol abuse, Wernicke’s encephalopathy, or thiamine deficiency. Use of prescription medication (including vaccines) within 30 days of the study start and non-prescription medication (including vitamin, mineral, and herbal supplements) within 14 days of the study start were prohibited. Participants were excluded if they were known to have hepatitis or tested positive for hepatitis or HIV, smoked > 10 cigarettes per day, or had a history of hypersensitivity to or intolerance of fluconazole or ondansetron.

### Study design and treatment

**Fig. 1:**
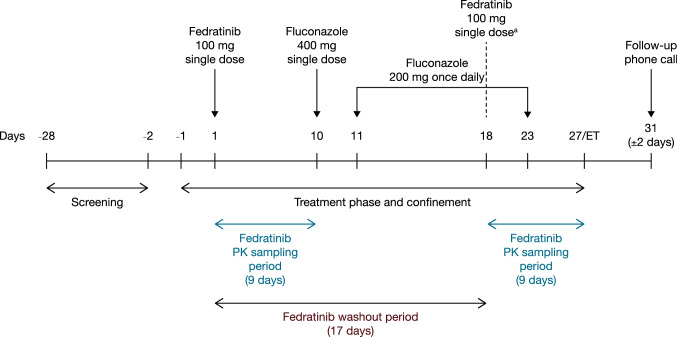
Overall study design. To reduce the potential for fedratinib-related nausea and vomiting, all participants received an oral dose of 8 mg ondansetron approximately 1 h before each fedratinib administration. Subsequent oral doses of ondansetron were given, as necessary, in accordance with USA prescribing information [27]. ^a^The day 18 fedratinib dose was administered concomitantly with the fluconazole dose. *ET* early termination

This was a non-randomized, fixed-sequence, open-label study consisting of a screening phase, treatment phase (including baseline), and follow-up telephone call (Fig. 1). The fixed-sequence crossover design of this study was selected to remove intersubject variability from comparison between treatments. Healthy participants were chosen to mitigate the potential confounding effects of disease state or other concomitant medications likely to be present in a population of patients with myelofibrosis.

A single-dose design was used for this study, as fedratinib exhibits linear and time-invariant PK in the clinically relevant dose range, which allows extrapolation to multiple doses [19]. The perpetrator drug chosen was fluconazole. The dosing regimen was selected following consideration of other fluconazole clinical DDI study designs that demonstrated significant DDI with fluconazole as perpetrator [25], with the aim of maximizing the inhibitory effects on CYP3A4 and CYP2C19 while limiting exposure to the agent. Continued dosing of fluconazole for an additional 5 days after fedratinib ensured that the maximum enzyme inhibitory effects were maintained during the fedratinib sampling time [25]. Fedratinib is not expected to have a clinically meaningful impact on the PK of fluconazole, which is mainly excreted unchanged into the urine and is unlikely to be affected by CYP pathways [26].

Participants resided at the clinical site from day − 1 to day 27. A single oral dose of fedratinib 100 mg was administered under fasted conditions on day 1. Participants received fluconazole 400 mg on day 10 and fluconazole 200 mg once daily on days 11–23. On day 18, participants received a single oral dose of fedratinib 100 mg, under fasted conditions, with fluconazole 200 mg. No food or beverages (except water) were allowed for at least 4 h after dosing; water was allowed as desired except for 1 h before and 1 h after each dose. Participants received ondansetron 8 mg by mouth approximately 1 h before each fedratinib administration to reduce the potential for fedratinib-related nausea and vomiting, with the option of a subsequent oral dose(s) of ondansetron as necessary [27]. Fluconazole is not expected to have a clinically meaningful impact on the PK of ondansetron as ondansetron is metabolized by multiple enzymes including CYP1A1/2, CYP3A4, and CYP2D6 [28]. Moreover, no clinically significant change to the area under the plasma concentration–time curve (AUC) of ondansetron was observed when coadministered with the moderate CYP3A inhibitor aprepitant [29]. Participants were discharged from the clinical site on day 27 and received a follow-up telephone call 4 days (± 2 days) after discharge. In the event that participants discontinued the study for any reason, an early termination (ET) visit was performed. Only safety assessments scheduled for the day of discharge were performed at the ET visit. Participants who discontinued the study also received a follow-up telephone call 4 days (± 2 days) after discharge. Participants who discontinued the study due to inclement weather returned to the clinic to complete the ET visit at a later date.

### PK sampling times and bioanalytical methods

On days 1 and 18, blood samples for determination of fedratinib plasma concentrations were collected before each dose and 0.5, 1, 1.5, 2, 3, 4, 6, 8, 12, 24, 48, 72, 120, 168, and 216 h post-dose. Plasma fedratinib concentrations were measured using a validated liquid chromatography–tandem mass spectrometry assay with a lower limit of quantification (LLOQ) of 1.00 ng/mL [19].

### Pharmacokinetic variables

Plasma PK parameters were calculated using noncompartmental methods. The PK parameters determined for fedratinib were maximum observed plasma concentration (*C*_max_), time to *C*_max_ (*T*_max_), AUC from time 0 to the time of the last quantifiable concentration (AUC_0–t_), AUC from time 0 to infinity (AUC_0–∞_), terminal elimination *t*_1/2_, apparent total plasma clearance (CL/F), and apparent total volume of distribution during the terminal phase (Vz/F).

### Statistical considerations

All participants who received at least one dose of fedratinib were included in the safety analyses; those with ≥ 1 dose of investigational product and one measurable concentration value were included in the PK analyses. The precision in the comparison of PK parameters, represented by the 90% confidence interval (CI) of the geometric mean ratios, was calculated for a range of potential sample sizes and values for intrasubject standard deviation (SD). Based on a previous study [30], the estimated intrasubject *C*_max_ SD for this study was 0.191. Twelve participants would provide adequate precision, estimated at 15.8%. To compare fedratinib PK parameters following single-dose administration in the presence and absence of fluconazole once daily, an analysis of variance (ANOVA) model with treatment as a fixed effect and participant as a random effect was performed on the natural log-transformed *C*_max_, AUC_0–t_, and AUC_0–∞_. The geometric means along with ratios of the geometric means and associated 90% CIs were calculated for the PK parameter comparison of fedratinib plus fluconazole (test) versus fedratinib alone (reference). For *T*_max_, Wilcoxon signed-rank test, Hodges–Lehmann estimate, and 90% CIs were calculated for the median difference between treatments. All statistical analyses were conducted using SAS Version 9.4 software (SAS Institute, Inc, Cary, NC) and Phoenix WinNonlin Version 8.0 software (Certara USA, Inc, Princeton, NJ).

### Safety assessment

Safety assessments included adverse event (AE) monitoring, review of concomitant medications and procedures, physical examinations, vital signs measurements, 12-lead ECGs, and clinical laboratory safety tests. All AEs were recorded from the time of informed consent until study completion, and up to 30 days after the last dose of fluconazole if reported to the investigator.

## Results

### Participants and participant disposition

Of the 29 healthy participants enrolled in the study, 16 (55.2%) completed the study as planned and were included in the PK population. A total of 13 (44.8%) participants discontinued treatment and withdrew from the study. Inclement weather resulting in a power outage in February 2021 led to discontinuation of 10 participants and sample temperature excursions, which resulted in exclusion of these samples from the PK analysis. Other causes of discontinuation were AEs (2 participants), and withdrawal by participant (1 participant). The study was ongoing during the COVID-19 pandemic (study period Jan 12, 2021, to May 9, 2021). All external guidelines published in the USA were adhered to and the pandemic did not affect safety results or planned study objectives. In the safety population, there were 25 male and 4 female participants enrolled; mean age was 39.8 years (range 19–64 years) and mean BMI was 26.9 kg/m^2^. The majority of participants were White (20/29, 69.0%) and not Hispanic or Latino (19/29, 65.5%) (Table 1). Participant demographics and baseline characteristics were similar between the safety and PK populations.

**Table 1:** Demographics and baseline characteristics

Characteristic	Safety population (*N* = 29)	PK population (*N* = 16)
Age, mean (range), years	39.8 (19–64)	37.4 (19–64)
Sex, *n* (%)		
Male	25 (86.2)	14 (87.5)
Female	4 (13.8)	2 (12.5)
Race, *n* (%)		
White	20 (69.0)	12 (75.0)
Black or African American	7 (24.1)	3 (18.8)
Asian	2 (6.9)	1 (6.3)
Ethnicity, *n* (%)		
Not Hispanic or Latino	19 (65.5)	11 (68.8)
Hispanic or Latino	10 (34.5)	5 (31.3)
Weight, mean (range), kg	80.37 (54.9–106.2)	78.98 (54.9–104.7)
Height, mean (range), cm	172.50 (156.6–186.4)	173.24 (156.6–186.4)
BMI, mean (range), kg/m^2^	26.87 (18.2–32.1)	26.18 (18.2–31.6)

BMI, body mass index

### Effect of fluconazole on fedratinib PK

#### Plasma concentration

**Fig. 2:**
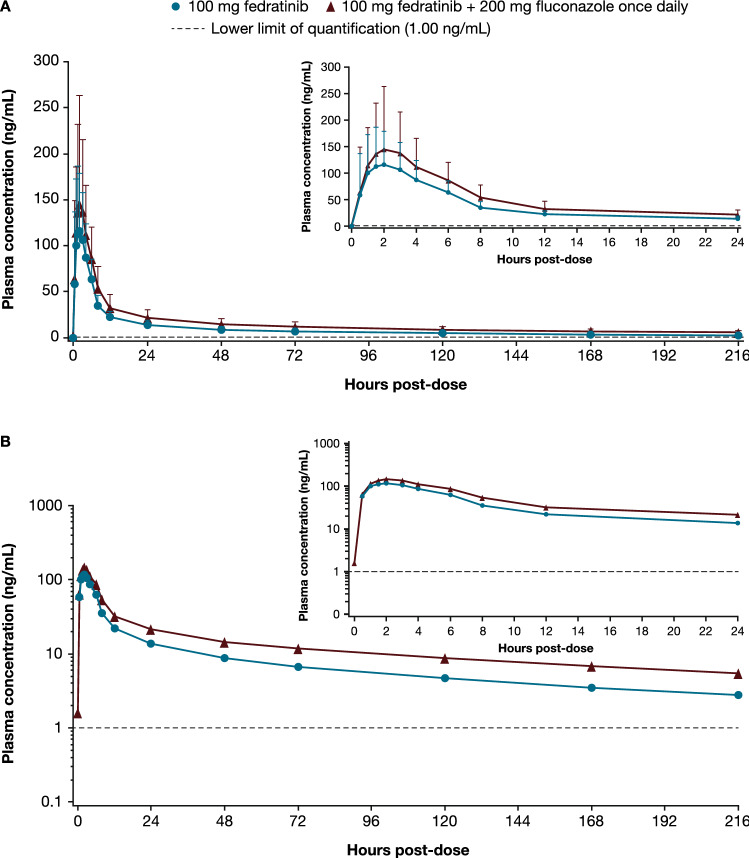
Mean plasma fedratinib concentration–time profiles following single oral doses of fedratinib alone or in combination with fluconazole. Mean (+ SD) fedratinib plasma concentration–time profiles displayed on **A** linear scale, **B** semi-logarithmic scale. For concentration values below the limit of quantification (ie, 1.00 ng/mL), a concentration value of zero was included for the computation of the arithmetic mean. If 50% or more of the values were below the limit of quantification at one time point, the arithmetic mean was reported as below the limit of quantification. The inlaid plot shows plasma concentration 0–24 h post-dose and main plot shows plasma concentration 0–216 h post-dose. Samples collected outside the protocol-defined window were excluded from calculation of summary statistics. *SD *standard deviation

Review of individual concentration–time data showed that, following both a single oral dose of fedratinib 100 mg on day 1 and a single oral dose of fedratinib 100 mg on day 18 with fluconazole 200 mg once daily, most participants had measurable fedratinib levels at the first sampling time (0.5 h after dosing). All participants had quantifiable concentrations of fedratinib from 1 h after dosing until the final sampling time (216 h after dosing) for both fedratinib with fluconazole and fedratinib alone (data not shown). Mean plasma concentration–time profiles for both fedratinib with fluconazole and fedratinib alone were characterized by a rapid absorption phase, with a median *T*_max_ of 2 h, after which fedratinib concentrations declined in a biphasic manner (Fig. 2).

#### PK parameters

PK parameters indicated higher exposure after a single oral dose of fedratinib 100 mg coadministered with fluconazole 200 mg once daily compared with fedratinib 100 mg alone (Table 2). Peak exposure, measured by geometric mean *C*_max_, was higher for fedratinib plus fluconazole compared with fedratinib alone (146 ng/mL vs 121 ng/mL). Geometric mean AUC_0–t_ was also greater for fedratinib plus fluconazole compared with fedratinib alone (2970 h·ng/mL vs 1900 h·ng/mL). After peak exposure, the plasma concentrations of fedratinib declined, with geometric mean *t*_1/2_ of 138 and 125 h, respectively, for fedratinib with fluconazole treatment and fedratinib alone. For five participants in the fedratinib plus fluconazole phase and seven participants in the fedratinib only phase, values for AUC_0–t_ were extrapolated to infinity (AUC_0–∞_) (geometric mean values of 4200 h·ng/mL and 2360 h·ng/mL, respectively).

**Table 2:** PK parameters by treatment

PK parameter	Fedratinib 100 mg (*n* = 16)	Fedratinib 100 mg + fluconazole 200 mg once daily (*n* = 16)
*C*_max_, ng/mL	121 (52.2)	146 (53.5)
*T*_max,_ median (min, max), h	2.00 (0.50, 4.02)	2.00 (1.02, 6.00)
AUC_0–t_, h·ng/mL	1900 (31.5)	2970 (34.8)
AUC_0–∞_, h·ng/mL^a^	2360 (21.9)	4200 (40.2)
*t*_1/2_, *h*	125 (22.1)	138 (27.2)
CL/F, L/h^a^	42.3 (21.9)	23.8 (40.2)
V_z_/F, L^a^	6500 (18.6)	3560 (34.0)

Data are geometric mean (geometric CV%) unless otherwise noted

^**a**^For AUC_0–∞_, CL/F, and V_z_/F, n = 7 for 100 mg fedratinib and n = 5 for fedratinib 100 mg + fluconazole 200 mg once daily; estimates were excluded from descriptive statistics where percent AUC extrapolated was > 20%

AUC, area under the plasma concentration–time curve; AUC_0–∞_, AUC from time 0 to infinity; AUC_0–t_, AUC from time 0 to the time of the last quantifiable concentration; CL/F, apparent total plasma clearance; C_max_, maximum observed plasma concentration; CV, coefficient of variation; PK, pharmacokinetic; t_1/2_, terminal elimination half-life; T_max_, time to maximum observed plasma concentration; V_z_/F, apparent volume of distribution during the terminal phase

Statistical analysis showed higher systemic exposure (*C*_max_ and AUC_0–t_) to fedratinib following fedratinib 100 mg plus fluconazole 200 mg once daily compared with a single oral dose of fedratinib 100 mg (Table 3). The ratio (%) of geometric least squares (LS) means between fedratinib 100 mg plus fluconazole 200 mg once daily (test) and fedratinib 100 mg (reference) was 156% for AUC_0–t_ and 121% for *C*_max_; intrasubject coefficients of variation were 11.6% and 18.5%, respectively. Due to the small sample size for AUC_0–∞_, no conclusion could be drawn regarding this parameter. There was no significant difference in *T*_max_ between fedratinib with fluconazole and fedratinib alone; median difference (90% CI) was 0.5 h (0.00–1.00; *p* = 0.156).

**Table 3:** Statistical comparisons of plasma PK parameters

PK parameter	Treatment	*N*	*n*	Geometric mean	Ratio (%) of geometric LS means, test/reference (90% CI)	Intrasubject CV (%)^a^
AUC_0–t_, h·ng/mL	100 mg fedratinib + 200 mg fluconazole once daily (test)	16	16	2970	156 (145, 168)	11.6
	100 mg fedratinib (reference)	16	16	1900		
AUC_0–∞_, h·ng/mL	100 mg fedratinib + 200 mg fluconazole once daily (test)	16	5	4100	170 (143, 202)	11.2
	100 mg fedratinib (reference)	16	7	2410		
*C*_max_, ng/mL	100 mg fedratinib + 200 mg fluconazole once daily (test)	16	16	146	121 (108, 135)	18.5
	100 mg fedratinib (reference)	16	16	121		

AUC_0–∞_ estimates were excluded from the statistical analysis where percent AUC extrapolated was > 20%. Geometric means, ratio, and 90% CI of the ratio of geometric means are from an ANOVA model with treatment as fixed effect and subject as a random effect on the natural log-transformed PK parameters

^a^Intrasubject CV (%) was defined as square root of [exp(MSE within subject ANOVA) – 1] × 100

ANOVA, analysis of variance; AUC, area under the plasma concentration–time curve; AUC_0–∞_, AUC from time 0 to infinity; AUC_0–t_, AUC from time 0 to the time of the last quantifiable concentration; C_max_, maximum observed plasma concentration; CV*,* coefficient of variation; LS*,* least squares; MSE*,* mean squared error; N*,* number of participants for each treatment; n, number of participants with evaluable values; PK, pharmacokinetic

#### Safety

The safety population included all 29 participants enrolled in the study. Of them, two participants discontinued the study due to treatment-emergent adverse events (TEAEs) (1 due to COVID-19 disease and 1 due to ventricular extrasystoles). Both events were considered mild in severity. TEAEs (*n* = 8) were experienced by 6 of 29 (20.7%) participants: 3 (10.3%) during treatment with fedratinib 100 mg alone, 2 (7.4%) during treatment with fluconazole 200 mg alone, and 1 (6.3%) during treatment with fedratinib 100 mg plus fluconazole 200 mg. The 8 TEAEs experienced were ventricular extrasystoles, abdominal discomfort, COVID-19 infection, tooth fracture, rhabdomyolysis, headache, oropharyngeal pain, and contact dermatitis. All were considered resolved by the end of the study, except ventricular extrasystoles (outcome unknown) and tooth fracture (not resolved). No TEAEs were suspected of being related to any study drug, and no deaths or other serious adverse events were reported. With the exception of ventricular extrasystoles, rhabdomyolysis, and contact dermatitis, which were reported in one participant each, no other clinically meaningful changes were observed in laboratory tests, physical examinations, or 12-lead ECG parameters.

## Discussion

This non-randomized, fixed-sequence, open-label study found that repeated doses of fluconazole increased systemic exposure to a single oral dose of fedratinib, with a 21% increase in *C*_max_ and 56% increase in AUC_0–t_ for a single oral dose of fedratinib coadministered with fluconazole compared with a single oral dose of fedratinib alone. Single oral doses of fedratinib 100 mg with or without fluconazole were well tolerated by the healthy adult participants in this study. Overall, the COVID-19 pandemic did not impact the ability to monitor and manage participant safety during the conduct of the study or affect the safety results and safety profile of fedratinib. All planned study objectives were achieved despite the pandemic.

PK samples were collected up to 216 h post-dose to cover more than approximately five times the effective *t*_1/2_ (~ 40 h) of fedratinib previously observed in healthy participants and patients with myelofibrosis [13, 31]. A 17-day washout period between reference and test administrations was used in this study. All plasma samples tested for fedratinib were below or close to the LLOQ (< 5% *C*_max_) before the fedratinib dose on day 18 (data not shown); this indicates that, given significant differences in terminal versus effective *t*_1/2_, the washout period based on five times the effective *t*_1/2_ was adequate. A similar washout period also appeared sufficient in other DDI studies with CYP3A4 modulators [31, 32]. Collectively, these results indicate that effective *t*_1/2_ is more useful in determining the PK sampling time and washout period for fedratinib than the terminal elimination *t*_1/2_. Single fedratinib doses of up to 680 mg have been administered in healthy participants, with doses up to 500 mg having been generally well tolerated [33]. The fedratinib dose of 100 mg was selected in this study so that fedratinib exposure, after the conservatively predicted up to fourfold increase by a dual CYP2C19 and CYP3A4 inhibitor [17], remained in the therapeutic range and did not exceed that from a 680-mg dose in healthy participants, assuming that fedratinib exposure increases in a linear direction for doses 100 mg and above [19, 33].

Current guidelines recommend avoiding concomitant treatment with fedratinib and dual CYP2C19 and CYP3A4 inhibitors, such as fluconazole [13, 14]. Previous studies have shown that coadministration of fedratinib with CYP3A4 inhibitors increases fedratinib exposure. In healthy men, the strong CYP3A4 inhibitor ketoconazole increased fedratinib *C*_max_ by 1.93-fold and AUC_0–t_ by 3.20-fold when coadministered with a single dose of fedratinib 300 mg compared with fedratinib 300 mg alone [31]. As expected for a CYP3A4 substrate, CYP3A4 inducers rifampin and efavirenz have been shown to decrease fedratinib exposure in healthy adults [32]. The less than twofold increase in fedratinib exposure by fluconazole that was observed in this study is similar to the predicted interaction magnitudes of moderate CYP3A4 inhibitors in a PBPK simulation study [17]. The mild increase in fedratinib exposure observed in this study suggests that CYP2C19 may only play a minor role in the metabolism of fedratinib. The PBPK model will be updated based on this trial and other emerging data if deemed appropriate. The *CYP2C19* gene is polymorphic; however, the frequency of *CYP2C19* functional polymorphism (e.g., CYP2C19*2 and CYP2C19*3) is about 3–5% in White and Black populations [34]. Therefore, *CYP2C19* genotype is unlikely to confound the results of this study in which 15/16 (94%) participants in the PK population were White or Black/African-American. Several JAK inhibitors are metabolized by multiple CYP enzymes, and coadministration with fluconazole results in a 234% increase in exposure to JAK2 inhibitor ruxolitinib [25]. The variance in the magnitude of exposure increase could be due to differences in the proportion of metabolism from CYP3A4 and CYP2C19 (CYP2C9 for ruxolitinib). Median *T*_max_ for fedratinib 100 mg was not significantly affected by coadministration of fluconazole in this study. This aligns with results from coadministration of fedratinib with ketoconazole, which also showed no effect on *T*_max_ [31].

There was no significant difference in terminal *t*_1/2_ of fedratinib when administered alone or in combination with fluconazole, which is consistent with the findings from previously published DDI studies with CYP modulators [31, 32]. Furthermore, the terminal phase of fedratinib concentration–time profile is likely to reflect redistribution and not elimination, which is supported by the large peripheral volume of distribution in the population PK model [19]. CL/F for fedratinib was lower in the presence of fluconazole, consistent with a decrease in V_z_/F.

The AUC_0–∞_ of several participants from both groups was removed from the statistical analysis due to percent values of AUC extrapolation higher than 20%. The contribution from extrapolation also appeared greater at doses of 100 mg compared with the contribution of extrapolation at higher doses. The intersubject variability of PK parameters such as *C*_max_ at the dose of 100 mg in this study (~ 52%) was low to moderate and appeared to be slightly larger than those reported (32–42%) at higher doses (≥ 300 mg) in previous studies with similar sample sizes (*n* = 12–16) [30, 32, 35, 36].

As in previous studies investigating the coadministration of fedratinib with CYP3A4 inhibitors, there were no serious or severe TEAEs reported. All TEAEs experienced by 6 of 29 participants were considered mild, and none were suspected of being related to any study drug.

## Conclusion

This study showed that systemic exposure to a single oral dose of fedratinib was mildly increased (by up to 56%) when fedratinib was coadministered with repeated doses of fluconazole. Single oral doses of fedratinib 100 mg administered both with and without fluconazole were well tolerated by healthy adult participants.

## Data Availability

Bristol Myers Squibb policy on data sharing may be found at https://www.bms.com/researchers-and-partners/independent-research/data-sharing-request-process.html.
